# The unrecognized potential of potential‐based achievement goals

**DOI:** 10.1111/bjep.12728

**Published:** 2024-12-14

**Authors:** Dirk Tempelaar

**Affiliations:** ^1^ Department of Quantitative Economics, School of Business and Economics Maastricht University Maastricht The Netherlands

**Keywords:** achievement goals, growth‐oriented goals, intrapersonal goals, potential‐based goals

## Abstract

**Background:**

For over a decade, growth‐oriented achievement goal constructs like potential‐based goals and personal best goals have remained relatively unnoticed. This empirical study aims to highlight that goal theorists might be limiting themselves by not incorporating potential‐based goals into their frameworks.

**Aims:**

The primary objective of this research was to underscore the significant yet underappreciated role of potential‐based goals in empirical studies within the 3 × 2 achievement goal framework.

**Samples:**

The sample comprises 10,079 international undergraduate students from a Dutch university, drawn from nine cohorts spanning academic years from 2015/2016 to 2023/2024.

**Methods:**

To validate the eight‐factor measurement model of achievement goals, we employed first and second‐order confirmatory factor analyses. Correlational analysis and structural equation models were utilized to explore the relationships between achievement goal measures, various learning dispositions, and academic performance.

**Results:**

Our analysis shows that all eight goal constructs clearly distinguish and confirm both first‐order and second‐order factor analysis models based on approach and avoidance factors. Further analyses include obtaining correlations and structural equation prediction models, where goal setting facets predict learning mindsets, such as intelligence theories, effort beliefs, autonomous and controlled regulation, motivation, engagement, learning approaches, and performance strategies.

**Conclusions:**

Within a system of criterion‐referenced grading of courses, our findings indicate that potential‐based goals play a pivotal role in exploring relationships with other learning dispositions and predicting performance. It is therefore imperative to incorporate these goals into our measurement instruments for goal frameworks, even if it means prioritizing them over other types of intrapersonal goals.

## INTRODUCTION

This journal is recognized as the foundational source for two future‐based achievement goal setting instruments designed for educational research. In 2006, Martin (2006) introduced Personal Bests (PBs), which stand out from other achievement goal setting approaches due to their emphasis on growth. Personal best goals are described as ‘specific, challenging, competitively self‐referenced targets towards which students strive to match or better a previous best’ (Martin, 2006, 2011b). Nearly a decade later, Elliot et al. (2015) introduced potential‐based goals as another expression of growth‐oriented achievement goal‐setting behaviour. The potential‐based goal constructs are the future‐oriented counterparts of the intrapersonal or self‐based goal constructs, that together with the task‐based and other‐based goal constructs shape the 3 × 2 achievement goal framework (Elliot, 1999, 2005; Elliot et al., 2011; Elliot & Murayama, 2008). While intrapersonal goals in instruments like the Achievement Goal Questionnaire (AGQ), (Elliot et al., 2011) are traditionally rooted in comparisons to past performance, potential‐based goals aim to capture similar performance aspects but with a future‐oriented perspective.

Despite the conceptual distinction from past‐based intrapersonal achievement goals and the evident advantages of integrating future perspectives of intrapersonal goals into goal‐setting frameworks, the utilization of growth‐oriented goal frameworks in empirical educational research has been relatively limited. Therefore, the primary aim of this paper is to illustrate that such neglect is unwarranted. We argue that potential‐based goals offer a well‐defined approach to achievement goals that not only clearly differentiate from other definitions but also provide significant predictive power in understanding relationships with other individual difference constructs relevant to achievement settings and performance.

## POTENTIAL‐BASED ACHIEVEMENT GOALS

The increased interest in educational psychology in students' growth orientation as a trigger for learning has been instrumental in the emergence of growth goals, among other growth directed learning dispositions (Martin, 2015). The development of the PBs scale in 2006 (Martin, 2006) was one of the earliest steps to measure growth goals, and it has since been utilized in empirical research (Martin, 2011b, 2015). Potential‐based goals (Elliot et al., 2015) evolved from achievement goal theory, with the 2 × 2 standards model of achievement goals (Elliot & Murayama, 2008) serving as its foundation. This model conceptualizes achievement goals using two components of competence: definition and valence. Definition refers to the standard used to assess competence, either task−/self‐based (mastery) or other‐based (performance). Valence indicates whether the focus is on the positive possibility of success (approach) or the negative possibility of failure (avoidance). Together, these components form the four goals of the 2 × 2 model. This foundation was extended to the 3 × 2 framework, which encompasses task‐based, intrapersonal, and interpersonal achievement goals (Elliot et al., 2011). In this framework, competence is defined in terms of task, oneself, and others. Task and self‐based intrapersonal goals represent mastery goals, while other‐based interpersonal goals represent performance goals. In the AGQ instrument aligned with the 3 × 2 framework (Elliot et al., 2015), all self‐based goal items traditionally referenced past performance. For instance, an item such as ‘My goal is to perform better on the exam in this class than I have done in the past on these types of exams’ (Elliot et al., 2015) reflects this standard. However, a growth orientation introduces an alternative standard focusing on proving one's personal potential, leading to a future‐oriented version of intrapersonal goal setting. For example, the potential‐based version of the aforementioned item becomes ‘My goal is to do as well as I can possibly do on the exams in this class’ (Elliot et al., 2015). As is clear from the item examples, the AGQ instrument including the future‐based goal items focus on examinations, in contrast to personal‐best goals, that refer to achievement in a more general perspective.

Elliot et al. (2015) suggest examining valence‐based dispositional variables as potential antecedents and consequences of potential‐based goals. All facets of adaptive motivation and engagement are expected to positively relate to goal setting, particularly the approach dimension (Elliot et al., 2011; Elliot & Hulleman, 2018). In a recent study, Bostwick et al. (2024) define a student's general growth orientation as a combination of task‐based growth goals, self‐based growth goals, and growth mindsets.

Both past‐based and growth‐based goals distinguish between an approach dimension and an avoidance dimension. The complete instrument used in this study is available in Data S1. An example of an approach‐based item from the original version by Elliot et al. (2015) is provided above. An example of an avoidance‐based item rephrased to suit our context is: ‘To avoid doing worse on the exam and quizzes than I normally do on these types of tests’ for the past‐oriented version, and ‘To avoid doing poorly in comparison to my absolute best on the exam and quizzes’ for the growth‐oriented version.

### Learning Mindsets

Students' growth orientation, as introduced by Bostwick et al. (2024), is a comprehensive concept composed of various constructs. Growth implies the ability to adapt and is naturally linked to learning frameworks that differentiate between adaptive and less adaptive or maladaptive approaches. Notable examples include the Motivation and Engagement Wheel by Martin (2008), which distinguishes adaptive from maladaptive motivation and engagement, self‐determination theory (Ryan & Deci, 2020), contrasting autonomous and controlled motivation, and deep versus surface learning approaches (Vermunt, 1998). One of the earliest representations of growth orientation in educational psychology is the concept of implicit theories of intelligence (Dweck, 1999, 2006). In Dweck's belief system, students with a growth orientation have an incremental view, belief in the malleability of their intelligence. In contrast, students with en entity view see intelligence as fixed (Dweck, 1999, 2006). Achievement goal theorists anticipate an antecedent role of implicit theories of intelligence for achievement goals (Elliot & Murayama, 2008; Norem, 2012). Entity theorists, perceiving intelligence as an inherent and stable trait, are inclined towards setting performance or interpersonal goals. Conversely, incremental theorists who believe intelligence can be enhanced through effort, prioritize mastery goals, including both task‐based and intrapersonal goals. Martin (2015), utilizing a cross‐lagged panel design to explore the dynamic interplay between implicit theories of intelligence and PBs goals, reached the somewhat surprising conclusion that growth goals appear to have a stronger directional influence compared to incremental and entity theories.

Mindsets extend beyond implicit theories. Dweck's monograph (1999) describes the functions and origins of the meaning system and its components: implicit theories of intelligence, effort beliefs, goal setting behaviour, achievement motivations and self‐regulation strategies. The two implicit theories are intertwined with beliefs about effort: believing in the utility of effort, as an incremental theorist, versus the futility of effort, as an entity theorist (Blackwell et al., 2007). Previous research by Tempelaar, Rienties, Giesbers, and Gijselaers (2015a) revealed that positive and negative beliefs about effort, stemming from implicit theories, mediate the relationships between implicit theories and goal‐setting behaviour. These findings are consistent with those of Blackwell (2002) and Blackwell et al. (2007). The evident significance of effort beliefs in relation to various motivational factors and academic success, independent of intelligence beliefs, is highlighted in Lavrijsen et al. (2022) and Boncquet et al. (2023). The research conducted by Lavrijsen et al. (2022), conceptually most similar to this study, reveals that the strongest bivariate relationships exist between intelligence beliefs and the avoidance dimensions of achievement goals, while the strongest bivariate relationships of effort beliefs are observed for the approach dimensions of achievement goals. It is an open question if this finding extends to potential‐based type of goals.

Expanding upon this, self‐determination theory (Ryan & Deci, 2020) introduces additional motivational concepts closely linked to students' mindsets: autonomous motivation, controlled motivation, and lack of motivation for regulating learning. Autonomously motivated students find their learning interesting or valuable, whereas those driven by controlled motivation engage in learning due to internal or external pressures (Boncquet et al., 2023; Lavrijsen et al., 2022). The research by Lavrijsen et al. (2022) further highlights that the strongest bivariate relationships are between controlled motivation and the avoidance dimensions of achievement goals, whereas the strongest bivariate relationships of autonomous motivation are with the approach dimensions of achievement goals. Again, it remains unclear whether this finding applies to potential‐based goals.

#### Motivation and engagement

The Motivation and Engagement Wheel framework (Collie & Martin, 2019; Martin, 2007), extends the distinction between adaptive and maladaptive learning beliefs to adaptive and maladaptive dimensions of motivation and engagement. The multidimensional model that synthesizes major conceptualizations of motivation and engagement from achievement goal theory, mindsets, self‐determination and expectancy‐value theory, postulates 11 factors of motivation and engagement that are subsumed under four higher‐order dimensions: adaptive motivation and engagement and maladaptive motivation and engagement. Adaptive cognitions and behaviours represent the mastery orientation of learning, whereas the maladaptive represent the performance orientation.

#### Student learning approaches

The distinction between adaptive and maladaptive approaches persists within the realm of student approaches to learning (SAL), as outlined by Biggs (1987). This dichotomy contrasts the deep approach, which seeks understanding, with the surface approach, which focuses on reproducing material. Typically, the adaptive‐maladaptive contrast governs the relationship between achievement goals and students' learning strategies in empirical research. For instance, Elliot et al. (1999) discovered that mastery approach goals relate positively with deep processing, while performance‐avoidance goals are positively associated with surface processing and negatively linked to deep processing. Performance‐approach goals, however, show a weaker connection to both processing types. Additional SAL frameworks, like Vermunt's (1998), introduce a third processing category: concrete or strategic processing, which emphasizes the transformation of new knowledge into tangible concepts and its practical application. Furthermore, Vermunt's framework posits three metacognitive learning regulation strategies: self‐regulation, external regulation, and lack of learning regulation. Vermunt and Donche (2017), summarizing empirical research utilizing this learning framework, note positive relations between mastery goal orientation and deep processing as well as self‐regulation of learning.

#### Academic achievement

Theoretical propositions suggest that achievement goals serve as predictors for performance metrics (Elliot et al. (2015); Elliot & Hulleman, 2018), albeit at moderate levels, with the most pronounced influence attributed to approach‐oriented achievement goals. Regarding potential‐based goals, Elliot et al. (2015) hold modest expectations: ‘*Relative to past‐based goals, potential‐based goals are more vague and abstract and provide a less concrete referent for guidance and feedback in self‐regulation. This would suggest that potential‐based goals may not be very powerful predictors of performance …*’ (p. 200). However, most empirical research on the relationship between goal‐setting behaviour and academic achievement comes from learning contexts with norm‐referenced grading systems. In our European context, where criterion‐referenced graded assessments are used, mastery‐based goals, as well as potential‐based goals, might have a greater impact compared to their role in studies with normative grading (Elliot, 2020, personal communication). It remains an open question how these two potentially opposing effects will influence the relationship between potential‐based goal setting and module performance.

#### Gender and prior education

Studies investigating gender effects on goal‐setting behaviour present divergent findings, with a general trend indicating that female students tend to be attracted towards mastery goals, while male students are more inclined towards performance‐approach goals (Linnenbrink‐Garcia et al., 2008). Martin (2015), in an analysis of PBs goals, notes that female students often demonstrate higher levels of such goals, as do students with higher levels of ability. Furthermore, exploring disparities in prior education represents an initial step in addressing the ‘ability confound hypothesis’ (Senko et al., 2011), which suggests that interpersonal goal setting correlates positively with performance due to the tendency for higher‐ability students to achieve higher scores on other‐based goals. While research specifically investigating the impact of prior education on goal‐setting behaviour is limited, it is plausible that the aforementioned observation regarding students with greater ability (Martin, 2015) may extend to differences in prior education, and that potential‐based goal setting may be affected in a similar manner as other goal facets.

#### Our research

Our primary research objective is to validate the 4 × 2 model of achievement goal orientation, which represents a notable expansion of the established 3 × 2 model (Elliot et al., 2011). We aim to show empirically that all eight factors within this model are distinguishable. Furthermore, beyond confirming internal validity, we endeavour to establish external validity. The first step in external validation is to compare potential‐based goal scores and other goal scores with the two available controls: prior education tier and gender. We expect that students from the advanced prior math tier and female students will achieve higher scores (Martin, 2015).

Although the potential‐based goals extension to the AGQ instrument is out for more than a decade, less than a handful empirical studies have investigated external validity. Specifically, we aim to demonstrate the importance of potential‐based goals in relation to both academic achievement and learning dispositions, next to other dimensions of goal orientation. We do this with the expectation that focusing on potential‐based goal pursuit will be positively associated with various adaptive learning dispositions and negatively associated with maladaptive learning dispositions.

Previous research on growth‐based goal setting by Elliot et al. (2015) and Martin (2015) utilized US and Australian samples. Our European context, which employs criterion‐referenced grading rather than norm‐referenced grading typical of Anglo‐Saxon classrooms, presents a different setting. Given the instrument's focus on exam performance, we expect this different context to influence outcomes such that approach‐type goals, both for potentially‐based goals and other goal facets, may dominate avoidance‐type goals in multivariate relationships with learning dispositions and, in specific, module performance. Additionally, potential‐based goals, along with other intra‐personal and inter‐personal goal perspectives, are expected to be dominated by task‐based mastery goals in multivariate relationships with module performance.

## METHODS

### Participants and educational context

This study examines first‐year students at a Dutch business and economics school from 2015/2016 to 2023/2024. The school stands out for its student‐centred approach, known as problem‐based learning (PBL), and its international orientation, offering programs primarily in English and attracting mostly international students. It holds Triple Crown accreditation, a rare distinction in global business schools. Among the 10,079 freshmen studied, 40% are female, 60% male, with 21% domestic and 79% international students. Most students enrol immediately after secondary school, typically aged 18–20 (19.20 average age, SD = .40), with data collected during the initial course: an introductory mathematics and statistics module.

The diverse national backgrounds of freshmen posed a challenge in assessing their prior education. However, most European high school systems, along with the International Baccalaureate system, distinguish between different tiers of mathematics education, each preparing students for various types of university studies: mathematics for arts and humanities, mathematics for social sciences, and mathematics for sciences. The second tier is required for admission to business and economics schools. In our cohorts, however, 36% of freshmen had completed education at the highest level, the third tier. Therefore, in this study, an advanced level of prior mathematics education was considered a relevant measure of previous educational background. National laws and regulations require all students to take the same curriculum and does not allow any form of streaming.

The international composition of our sample suggested that cultural differences among students could be a factor. Previous research, such as Daumiller and Zarrinabadi (2021), has argued that students from collectivistic cultures may exhibit different levels of goal‐setting behaviour compared to those from more individualistic cultures. However, initial analyses of our sample did not reveal any cultural differences of notable magnitude. The institution primarily employs PBL through small tutorial groups of 15 students each. To accommodate varying levels of proficiency, especially in mathematics and statistics, online resources supplement traditional methods, resulting in a blended learning environment. Students individually prepare tutorials with e‐learning resources, followed by collaborative problem‐solving in small groups, reflecting a ‘flipped classroom’ teaching approach (Non & Tempelaar, 2016; Williams et al., 2017).

To further support student learning in this module, Dispositional Learning Analytics (DLA) is applied. Learning Analytics (LA, Buckingham Shum & Deakin Crick, 2012) aims to furnish students with feedback on their learning progress based on trace data obtained from their interaction with digital learning platforms. Dispositional Learning Analytics (Buckingham Shum & Deakin Crick, 2012; Tempelaar et al., 2018; Tempelaar, Rienties, & Giesbers, 2015) augments this approach by incorporating additional data from self‐report surveys assessing learning dispositions. The dispositions surveys are based on social‐cognitive theories of learning and serve two purposes: providing personalized feedback and supplying individual datasets for statistical projects, enhancing statistical data analysis skills. Suboptimal learning strategies, along with issues like inadequate planning and poor study management—topics covered in early surveys—serve as examples of learning feedback, along with feedback derived from learning progress data. This dual utilization of survey data ensures high response rates and quality data, minimizing the tendency to provide ‘satisfying’ responses (Elliot & Hulleman, 2018), as students understand the importance of dataset variation for effective analysis. Therefore, the selection of survey instruments is guided by the aim of supporting student learning, rather than being specifically tailored to the design of this particular research. Data S2 contains sample items for all scales contained in the learning disposition instruments.

### Materials

The core instrument employed in this study is the expanded AGQ, which is based on the 3 × 2 achievement goal model (Elliot et al., 2011). This model encompasses six goal constructs, delineating three definitions—task, self, and other—and two valences: approach and avoidance. The AGQ extension involves discerning two sub‐dimensions within the intrapersonal or self‐dimension: past and potential (Elliot et al., 2015). To minimize the correlation between approach and avoidance goals, often observed in achievement goal measures, Elliot et al. (2015) implemented a two‐step assessment procedure. First, participants were asked whether they had adopted a specific goal. They only reported the strength of their adopted goals if they responded affirmatively, by answering the items related to those goals. In our study, we opted for the standard single‐step procedure, which directly measured the intensity of goal setting by administering the full AGQ instrument, in order to keep it concise. However, we included both aspects of the self‐dimension, resulting in eight distinct constructs: Task‐approach (TAP), Task‐avoidance (TAV), two interpersonal goals—Other‐approach (OAP) and Other‐avoidance (OAV)—and four intrapersonal goals: Self‐approach (SAP), Self‐avoidance (SAV), Potential‐approach (PAP), and Potential‐avoidance (PAV) achievement goals. Each goal facet was measured using three items (the complete instrument is available in the Data S1). The wording of the instrument items was adjusted to guide students towards the two module assessments. Specifically, the standard performance reference in AGQ‐items, ‘exams in this class,’ was replaced with ‘exam and quizzes in this class,’ to reflect the two performance standards pertinent to our module. The instrument employed a 7‐point Likert scale and was administered midway through the module, allowing students to familiarize themselves with the learning context and, criterion‐referenced graded, assessments.

Implicit theories were operationalized using a two‐dimensional approach, adopting measures of entity and incremental implicit theories of intelligence, as in Martin (2015). Dweck's (1999) Theories of Intelligence Scale – Self Form for Adults, comprises four items reflecting entity theory and four reflecting incremental theory. Effort beliefs were assessed using two distinct sources: Dweck (1999) and Blackwell (2002). Dweck (1999) contains various sample statements where exerting effort is perceived as reflecting either low ability or as a means to activate and enhance one's ability. Additionally, Effort belief items from Blackwell (2002) were employed, consisting of five positive and five negative items (refer also to Blackwell et al., 2007; Tempelaar et al., 2015a). Autonomous and controlled motivation were measured using the Academic Motivation Scale (AMS, Vallerand et al., 1992) The AMS prompts individuals to answer the question ‘Why are you attending college?’ and counts seven subscales, three intrinsic motivation and one identified motivation subscale, representing autonomous motivation, the two subscales introjected and external motivation representing controlled motivation, and the a‐motivation subscale denoting the absence of regulation.

The Motivation and Engagement Scale instrument (Martin, 2008, 2011a), based on Martin's (2007) Motivation and Engagement Wheel framework, categorizes learning cognitions and behaviours into adaptive and maladaptive types across cognitive and behavioural domains. Adaptive cognitive factors include Self‐belief, Value of school, and Learning focus, while Planning, Study management, and Persistence shape adaptive behavioural factors. Maladaptive cognitive factors are Anxiety, Failure avoidance, and Uncertain control, whereas Self‐sabotage and Disengagement represent maladaptive behavioural factors. Maladaptive cognitive factors can sometimes act as activating cognitions depending on context, unlike maladaptive behaviours, which are consistently deactivating.

Vermunt's (1998) student learning pattern (ILS) instrument was used to evaluate cognitive learning processing and metacognitive learning regulation strategies. The instrument includes five subscales for cognitive processing strategies, distinguishing between deep learning (Critical Processing, Relating and Structuring), step‐wise or surface learning (Analysing, Memorizing) and Concrete processing. Additionally, five subscales for metacognitive regulation strategies differentiate between self‐regulation (of learning processes and content), external regulation (of learning process and results), and lack of regulation. The assessment was conducted at the beginning of the academic study, indicating that students' typical learning patterns are those developed during high school education.

To summarize the timing of the learning disposition instruments: the AGQ goal‐setting instrument, which requires familiarity with the module content and assessments, was administered midway through the module. In contrast, all other disposition instruments were administered during the first week of the module, likely reflecting dispositions formed during high school education.

Assessment in this module includes three biweekly quizzes covering mathematics and statistics, alongside a comprehensive final exam. Quizzes serve primarily formatively, assessing intermediate learning objectives and encouraging students to monitor progress. Quiz scores combine into MathQuizzes and StatsQuizzes, while the final exam yields MathExam and StatsExam scores. To account for cohort variations, all assessment scores were transformed into z‐scores.

The contextual variables in the descriptive part of the analysis include Gender and Prior mathematics education (indicated by having completed an advanced mathematics tier). Gender data were obtained from the school's administrative system, while prior education data were self‐reported.

### Procedure

In the initial 8 weeks of their first academic semester, students completed two required modules: one integrating organizational theory and marketing, and another combining mathematics & statistics. In the first and fourth weeks, students filled out self‐report questionnaires on learning dispositions for a data‐analysis project, both in and outside class. Since it's their early university experience, responses to the disposition surveys likely reflect high school habits. The achievement goals questionnaire was given midway through to ensure students were familiar with the learning environment and assessments: at the end of the fourth week of our eight‐week module. Given the nature of the project, there were no missing values except for those resulting from students dropping out of the module, who were subsequently excluded from the study.

### Statistical analyses

Latent variable modelling was conducted using MPlus (version 8.7), while scale scores were modelled using SPSS (version 27). Due to the large sample size, evaluating statistical significance alone is insufficient; therefore, effect sizes are included as a measure of practical significance in the analysis. As per the approach outlined in Collie and Martin (2019), we utilize *R* ^2^ as effect size measure for prediction equations (.01, .09, and .25 for small, medium, and large effect sizes) and standardized beta estimates as effect size measure for individual predictors (*β* ≥ .05 is a small effect, *β* ≥ .10 is a medium effect, and *β* ≥ .25 is a large effect). A conservative benchmark of .001 is applied for statistical significance. The cross‐sectional nature of our disposition data prevents us from deriving structural equation models that allow for causal interpretation. Instead, we develop structural equation models as predictive models, representing relationships that predict learning dispositions or performance based on achievement goal constructs, without distinguishing between antecedence and consequence relationships. Specifically, when examining the relationship between learning mindsets and goal setting, one could argue for a reverse direction. However, the specification of goal setting predicting mindsets aligns with findings from Martin (2015). To align with the aim of predicting learning dispositions from goal‐setting behaviour and to remain consistent with previous research, we included contextual variables, such as gender and prior education, in the descriptive analysis of achievement goals, but not into the inferential modelling. Lastly, although some studies caution against the risk of (multi)collinearity, no evidence supporting this concern has been found in our analyses; correlations between scales are all .8 or lower, correlations between latent constructs are all below .9. All structural equation prediction models have been estimated in two versions: one with all eight goal constructs as predictors (left panel in the tables) and another with only the two potential‐based goal constructs as predictors (right panel in the tables). This approach allows us to understand the unique and combined contributions of potential‐based goals in predicting dispositions and module performance.

## RESULTS

### Descriptives and confirmatory factor analysis

The initial objective is to analyse achievement goal data independently and juxtapose the findings with those delineated in the two empirical studies outlined by Elliot et al. (2015). Descriptive statistics and the reliabilities of the eight achievement goal scale scores are presented in Table 1. The means of task‐based and intrapersonal (self and potential) achievement goals all surpass the neutral score of four, aligning with the outcomes identified by Elliot et al. (2015). Interpersonal achievement goals exhibit slightly lower scores but remain above the neutral score. Consequently, there is greater variation in other‐based scores compared to the other metrics, which tend to manifest ceiling effects. All goal scales demonstrate robust internal consistency, surpassing those documented in Elliot et al. (2015). Intraclass correlations (ICC), nesting students in cohorts, are all below .01, and none of the random intercepts for cohorts reaches statistical significance.

**TABLE 1:** Descriptive statistics and composite reliabilities.

	M	SD	Observed range	Omega	ICC
TAP: Task‐approach goals	6.280	.823	1.00–7.00	.854	.173%
TAV: Task‐avoidance goals	6.154	.927	1.00–7.00	.781	.073%
SAP: Self‐approach goals	5.804	.982	1.00–7.00	.793	.024%
SAV: Self‐avoidance goals	5.809	1.018	1.00–7.00	.797	.151%
OAP: Other‐approach goals	4.368	1.481	1.00–7.00	.915	.669%
OAV: Other‐avoidance goals	4.798	1.435	1.00–7.00	.905	.509%
PAP: Potential‐approach goals	6.437	.758	1.00–7.00	.864	.175%
PAV: Potential avoidance goals	5.895	.971	1.00–7.00	.772	.133%

Correlations among the eight achievement goal scale scores are detailed in Table 2. An expanded version of Table 2 is included as Data S3, containing bootstrap 95% confidence intervals. Standard errors of most correlations fall in the [.011, .013] range, creating confidence intervals with a margin of error of about .024. Approach and avoidance aspects within all four definitions are distinctly discernible. Notably, regarding potential‐based goals, the correlation between approach and avoidance is dominated by the correlations with TAP, TAV, and SAV goals (with differences up to three standard errors in size), suggesting that a two‐step approach may not be necessary as proposed by Elliot et al. (2015). The largest VIF, variance inflation factor at scale level, refers to the relationship of approach and avoidance dimensions of the other‐based goals. That VIF equals 2.84, far below any benchmark for (multi)collinearity. The largest VIF referring to potential‐based goals is as low as 1.83.

**TABLE 2:** Correlations of achievement goal scale scores.

	TAP	TAV	SAP	SAV	OAP	OAV	PAP	PAV
TAP	1.000							
TAV	.721	1.000						
SAP	.604	.533	1.000					
SAV	.506	.613	.710	1.000				
OAP	.280	.224	.332	.287	1.000			
OAV	.261	.318	.314	.407	.805	1.000		
PAP	.627	.518	.528	.444	.170	.169	1.000	
PAV	.537	.620	561	.673	.324	.402	.592	1.000

*Note*: *p* < .001 for all correlations.

A first‐order confirmatory factor analysis allowing intercorrelations between all equally numbered items in the two valences of all four definitions (these items are mirrors, replacing each time the approach based formulation by an avoidance based formulation) showed good fit: *χ* ^2^(212) = 4054, *p* < .001, CFI = .977, TLI = .970, RMSEA = .042, 90% CI RMSEA = (.041, .044).

However, a more parsimonious model emerged as the second‐order CFA with the approach and avoidance valences as a second‐order factor; again, this aligns with the findings of Elliot et al. (2015), who determined that the second‐order approach‐avoidance model was superior to the first‐order general factor model. That second‐order approach‐avoidance model allows setting all first order latent factor correlations to zero except for the four‐factor correlations relating to the approach and avoidance dimensions of the same definition. The fit indices of that second‐order CFA are *χ* ^2^(227) = 5000, *p* < .001, CFI = .971, TLI = .965, RMSEA = .046, 90% CI RMSEA = (.045, .047). The second‐order factor explains most of the variation of the task‐based and self‐based achievement goals: squared multiple correlations for the structural equations are .748 and .752 for the approach and avoidance valences of task‐based goals, .736 and .813 for the approach and avoidance valences of self‐based goals and .605 and .902 for the approach and avoidance valences of potential‐based goals. Only the other‐based goals are less well explained by the second‐order factors: squared multiple correlations for the structural equations are .163 and .225 for the approach and avoidance valences. Indicating the congruency of all goal definitions except the inter‐personal ones: not only self‐based and potential‐based but also task‐based ones.

### Socio‐demographic factors: Gender and prior education

Significant gender disparities are evident across seven out of eight goal achievement metrics. Female students demonstrate superior performance to male students across all task and intra‐individual goal scores. Conversely, male students exhibit higher levels in the approach facet of inter‐individual goal scores, whilst for the OAV goal, the difference is not statistically significant. Particularly notable are the substantial gender discrepancies observed in the potential definition of achievement goals, as indicated in the left panel of Table 3.

**TABLE 3:** Gender (left panel) and prior education (right panel) differences in goal achievement scale scores.

	Female	Male	*t*‐value	*p*‐value	Effect size	Math major	Math minor	*t*‐value	*p*‐value	Effect size
TAP: Task‐approach goals	6.359	6.229	7.857	<.001	.159	6.337	6.255	4.908	<.001	.101
TAV: Task‐avoidance goals	6.287	6.066	12.097	<.001	.240	6.178	6.144	1.744	.081	.037
SAP: Self‐approach goals	5.861	5.766	4.756	<.001	.097	5.837	5.787	2.448	.014	.051
SAV: Self‐avoidance goals	5.903	5.748	7.543	<.001	.153	5.815	5.807	.384	.701	.008
OAP: Other‐approach goals	4.272	4.433	−5.368	<.001	−.108	4.549	4.266	9.143	<.001	.191
OAV: Other‐avoidance goals	4.838	4.773	2.243	.012	.045	4.924	4.726	6.594	<.001	.138
PAP: Potential‐approach goals	6.570	6.349	15.072	<.001	.295	6.473	6.425	3.088	.002	.064
PAV: Potential avoidance goals	6.021	5.813	10.806	<.001	.215	5.935	5.874	2.991	.003	.063

Prior education differences in goal achievement scores are much less pronounced. Employing a stringent significance threshold of .001, students who pursued advanced mathematics courses outperform their counterparts from other tracks in three out of eight achievement goal scales, as depicted in the right panel of Table 3: specifically, the two other‐oriented goals and the TAP goal, with non‐significant differences for the remaining goal scores. Differences in the approach dimensions between students with varying competence levels tend to be marginally larger than disparities in the avoidance dimensions, aligning with anticipated patterns in self‐perceptions of competence (Elliot & Hulleman, 2018; Elliot & Murayama, 2008). The relatively substantial discrepancy in other‐oriented goals favouring high‐ability students introduces the potential for ability confounds in the interpersonal goal performance relationship.

**FIGURE 1:**
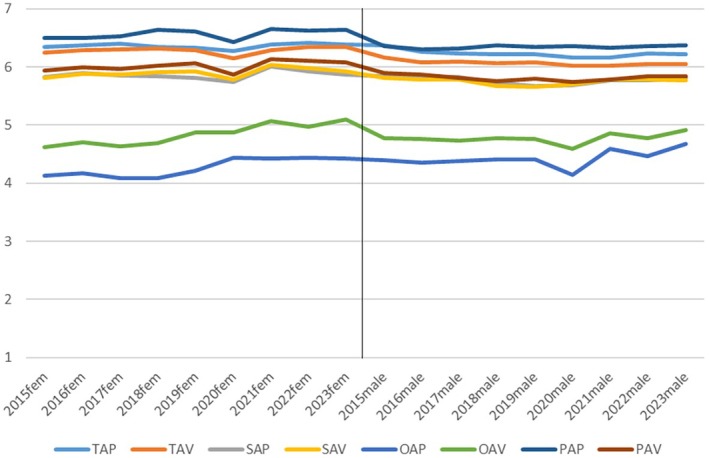
Goal achievement scale scores over time, for female (left) and male (right) students.

As was also evident from the very low values of intraclass correlations, goal setting exhibits notable stability over time, as depicted in Figure 1, with data for female students presented in the left panel and data for male students in the right panel. Formal assessments for differences across years yield predominantly insignificant results, with effect sizes all below 1%; therefore, these results will not be detailed here. The only deviation from this consistent pattern is observed in the levels of the two other‐oriented goals, where a slight increase can be discerned during the Covid‐19 and post‐Covid years.

### Achievement goals and implicit theories, effort beliefs and academic motivations

Bivariate correlations of achievement goals measures with implicit theories, effort beliefs and academic motivations are contained in Table 4. An expanded version of Table 4 is included as Data S3, containing bootstrap 95% confidence intervals. Standard errors of most correlations fall in the [.013, .014] range, creating confidence intervals with a margin of error of about .027. Consistent with our prior research findings (Tempelaar et al., 2015a), effort beliefs exhibit stronger associations with achievement goals compared to implicit theory measures. Specifically, Effort positive beliefs demonstrate stronger correlations than incremental view scores with all eight facets of achievement goals, while Effort negative beliefs exhibit stronger correlations (in absolute terms) than entity view scores with all statistically significant facets of achievement goals. Predictions related to mastery and performance goals (Norem, 2012) are not confirmed: mastery goals are indeed positively related to the incremental view and negatively related to the entity view, but performance goals are unrelated to both views. Another pattern emerges from the data in Table 4: approach dimensions demonstrate stronger correlations with the incremental view and positive effort beliefs than their corresponding avoidance dimensions across all definitions of mastery goals. Additionally, all approach dimensions exhibit stronger negative correlations with the entity view and negative effort beliefs compared to avoidance dimensions, again, across all definitions of mastery goals.

**TABLE 4:** Correlations of achievement goal scale scores with implicit theories, effort beliefs, and academic motivations, and composite reliabilities.

	TAP	TAV	SAP	SAV	OAP	OAV	PAP	PAV	Omega
Incremental Theory	.065***	.055***	.132***	.112***	.021*	.023*	.118***	.074***	.843
Entity Theory	−.049***	−.037***	−.074***	−.033**	.014	.023*	−.099***	−.051***	.863
Effort positive	.172***	.141***	.187***	.141***	.107***	.099***	.228***	.187***	.628
Effort negative	−.156***	−.106***	−.096***	−.065***	−.040*	.007	−.186***	−.121***	.690
Autonomous	.152***	.125***	.174***	.151***	.131***	.135***	.206***	.185***	.831
Controlled	.115***	.121***	.144***	.150***	.196***	.216***	.105***	.147***	.812
A‐motivation	−.192***	−.159***	−.122***	−.125***	−.027**	−.037***	−.200***	−.157***	.819

Note: *: *p* < .05; **: *p* < .01; ***: *p* < .001.

Both Autonomous and Controlled motivation types display positive correlations with all goal dimensions, with autonomous motivation showing its strongest relationships with the mastery goal facets and controlled motivation with the performance goal facets. Conversely, a‐motivation consistently demonstrates negative associations with goal facets.

The structural equation model predicting the two implicit theories entity and incremental view, the two effort beliefs positive and negative effort view, and the three academic motivations of autonomous and controlled motivation and a‐motivation, indicates good fit: *χ* ^2^(1792) = 18,665, *p* < .001, CFI = .938, TLI = .933, RMSEA = .033, 90% CI RMSEA = (.032, .033), SRMR = .036. The left panel of Table 5 presents standardized beta coefficients from the prediction model, encompassing predictors in structural relationships that exceed a statistical significance threshold of .001. Across all prediction equations except for controlled motivation, the potential‐based approach goal emerges as the dominant predictor, boasting larger beta coefficients, reaching medium‐sized effects, compared to any other goal facet. The exception is controlled motivation, which is primarily predicted by the two inter‐personal goal dimensions. In the multivariate context, avoidance goals contribute minimally to predictive power beyond approach goals, with the exception of the inter‐personal dimension. Prediction equation effect sizes range from small to medium; especially intelligence beliefs are poorly predicted.

**TABLE 5:** Standardized beta coefficients from the structural equation model predicting implicit theories, effort beliefs and academic motivations from achievement goals; left panel applying all goal constructs, right panel potential‐based goals only.

	TAP	TAV	SAP	SAV	OAP	OAV	PAP	PAV	*R* ^2^	PAP	PAV	*R* ^2^
Incremental Theory	−.149		.133				.190		.038	.179	−.021	.027
Entity Theory	.106		−.090			.050	−.172		.027	−.182	.057	.022
Effort positive			.109		.066		.279		.146	.287	.111	.136
Effort negative	−.101				−.157	.191	−.201		.086	−.304	.037	.078
Autonomous			.092			.078	.206	.078	.137	.187	.213	.136
Controlled				.074	.084	.156	.071		.092	−.039	.273	.061
A‐motivation	−.129				.038		−.172		.075	−.227	−.049	.072

Note: *p* < .001 in left panel.

The right panel of Table 5 presents the structural equation model, limiting predictors to the two potential‐based goals. The crucial role of the approach dimension within the potential facet of goal pursuit is evident from the comparison of explained variation between the left and right panels. Except for Controlled motivation, the decrease in predictive power is relatively minor. The two constructs most effectively predicted by goal pursuit, the positive effort view and Autonomous motivation, show minimal loss in predictive power when predictors are restricted to potential‐based constructs.

### Achievement goals and motivation and engagement

The correlations outlined in Table 6 validate the anticipated positive bivariate connections between goal dimensions and adaptive motivation and engagement, while demonstrating negative correlations between goal dimensions and maladaptive engagement. An expanded version of Table 6 is included as Data S3, containing bootstrap 95% confidence intervals. Standard errors of all correlations fall in the [.010, .011] range, creating confidence intervals with a margin of error of about .021. However, a discernible pattern is lacking concerning the bivariate relationships between goal dimensions and maladaptive motivation. Anxiety displays predominantly positive associations with all types of goal setting, Uncertain Control exhibits negative correlations, and Failure Avoidance manifests a mixed pattern. Once more, with the exception of maladaptive motivation, potential‐based goals overshadow the other three goal definitions in terms of correlation magnitudes, both for approach and avoidance dimensions.

**TABLE 6:** Correlations of achievement goal scale scores with motivation and engagement scales, and composite reliabilities.

	TAP	TAV	SAP	SAV	OAP	OAV	PAP	PAV	Omega
Self‐belief	.212***	.155***	.190***	.158***	.159***	.127***	.221***	.203***	.780
Value of school	.203***	.178***	.196***	.177***	.109***	.128***	.243***	.213***	.667
Learning focus	.239***	.212***	.237***	.216***	.117***	.136***	.296***	.249***	.779
Planning	.134***	.109***	.134***	.101***	.098***	.085***	.214***	.163***	.783
Study manag.	.161***	.141***	.160***	.143***	.085***	.088***	.234***	.180***	.761
Persistence	.216***	.176***	.186***	.163***	.163***	.139***	.278***	.238***	.783
Anxiety	.016	.069***	.071***	.095***	−.052***	.029**	.064***	.064	.828
Failure avoid.	−.061***	−.027**	.001	.025*	.077***	.117***	−.102***	−.012	.845
Uncertain contr.	−.090***	−.039**	−.024*	.001	−.097***	−.030**	−.080***	−.056***	.806
Self‐sabotage	−.178***	−.141***	−.085***	−.069***	−.031**	−.018	−.239***	−.135***	.824
Disengagement	−.196***	−.163***	−.139***	−.130***	−.052***	−.061***	−.248***	−.281***	.722

Note: *: *p* < .05; **: *p* < .01; ***: *p* < .001.

The structural equation model predicting 11 adaptive and maladaptive motivation and engagement latent factors indicates good fit: *χ* ^2^(2072) = 20,096, *p* < .001, CFI = .948, TLI = .943, RMSEA = .029, 90% CI RMSEA = (.029, .031), SRMR = .032. In the multivariate analysis, the significance of the approach dimension of the potential‐based goal is evident, as it accounts for the majority of the explained variance in all six prediction equations of adaptive cognitions and behaviours, as well as both prediction equations of maladaptive behaviours, as shown in the left panel of Table 7. At predictor level, standardized beta estimates based effect sizes even reach large effects for the approach dimension of future goals, where at the prediction equation level, *R* ^2^ effect sizes range from small to medium.

**TABLE 7:** Standardized beta coefficients from the structural equation model predicting motivation and engagement scales from achievement goals; left panel applying all goal constructs, right panel potential‐based goals only.

	TAP	TAV	SAP	SAV	OAP	OAV	PAP	PAV	*R* ^2^	PAP	PAV	*R* ^2^
Self‐belief	.197	−.144			.202	−.086	.150	.112	.131	.202	.150	.106
Value of school	.079					.094	.321		.170	.277	.171	.171
Learning focus			.080			.078	.339		.176	.291	.161	.176
Planning	−.056				.077		.308		.085	.242	.050	.078
Study manag.					.052		.298		.097	.269	.054	.095
Persistence				−.121	.154		.325	.131	.172	.286	.129	.150
Anxiety	−.162	.182	.068	−.403	.345	−.142		.065	.029	.096	.014
Failure avoid.	−.071	−.036	.045	.036	−.008	.159	−.138	.023	.055	−.256	.170	.034
Uncertain contr.	−.157		.157		−.334	.246	−.080		.053	−.128	.018	.013
Self‐sabotage		−.162		.212			−.329		.118	−.366	.067	.104
Disengagement	−.105		.090				−.292	−.046	.124	−.303	−.061	.121

Note: *p* < .001 in left panel.

The comparison of explained variation between the left and right panels of Table 7, where predictors are limited to potential‐based goals, highlights the crucial role of the approach dimension in potential goal pursuit. With the exception of the two maladaptive cognitions, Anxiety and Uncertain control—which are best predicted by interpersonal goal setting—the decrease in explained variation is small or non‐existent.

The prediction equations presented in Table 7 reveal modest associations for the three maladaptive cognitive factors. In this context, potential‐based goals play a secondary role in prediction, with other‐based goal dimensions overtaking predictive power.

### Achievement goals and cognitive learning processing and metacognitive learning regulation strategies

In bivariate relationships between learning approach scales and goal setting behaviour, correlations of approach goals tend to dominate corresponding correlations of avoidance goals. Correlations of potential‐based goal setting dominate correlations of other goal dimensions for both surface approach of learning scales Analysing and Memorizing and the deep approach of learning scale Relating & Structuring: see Table 8. A similar directed dominance can be observed among the two scales of External Regulation: of Process and Results. An expanded version of Table 8 is included as Data S3, containing bootstrap 95% confidence intervals. Standard errors of all correlations fall in the [.010, .011] range, creating confidence intervals with a margin of error of about .021.

**TABLE 8:** Correlations of achievement goal scale scores with learning approach scales, and composite reliabilities.

	TAP	TAV	SAP	SAV	OAP	OAV	PAP	PAV	Omega
Critical Proc.	.075***	.046***	.106***	.082***	.148***	.124***	.075***	.108***	.663
Relating & Struc.	.136***	.105***	.143***	.114***	.130***	.123***	.168***	.164***	.771
Concrete proc.	.108***	.073***	.154***	.113***	.135***	.120***	.115***	.125***	.680
Analysing	.154***	.123***	.179***	.141***	.141***	.121***	.206***	.180***	.684
Memorizing	.101***	.091***	.118***	.101***	.101***	.097***	.146***	.128***	.740
Self‐Reg. Proc.	.097***	.074***	.152***	.107***	.121***	.106***	.151***	.139***	.723
Self‐Reg. Cont.	.063***	.058***	.139***	.104***	.144***	.117***	.112***	.125***	.662
Ext.‐Reg. Proc.	.120***	.116***	.136***	.133***	.103***	.112***	.163***	.141***	.540
Ext.‐Reg. Results	.198***	.167***	.161***	.159***	.103***	.121***	.204***	.182***	.542
Lack Regulation	−.070***	−.029**	.014	.024*	−.042***	.005	−.076***	−.033***	.725

Note: *: *p* < .05; **: *p* < .01; ***: *p* < .001.

The structural equation model predicting five cognitive learning processing strategies and five metacognitive learning regulation strategies, indicates good fit: *χ* ^2^(2881) = 30,849, *p* < .001, CFI = .907, TLI = .901, RMSEA = .031, 90% CI RMSEA = (.031, .031), SRMR = .038. Effect sizes of prediction equations range from small to medium. Approach dimension of goal setting, and among them, the potential‐based approach goal, account for most of the predictive power, with medium‐sized β‐based effect sizes; avoidance dimensions limit their role to predicting external regulation and lack of regulation of learning: see Table 9, left panel.

**TABLE 9:** Standardized beta coefficients from the structural equation model predicting learning approach scales from achievement goals; left panel applying all goal constructs, right panel potential‐based goals only.

	TAP	TAV	SAP	SAV	OAP	OAV	PAP	PAV	R^2^	PAP	PAV	R^2^
Critical Proc.			.115		.181				.062	−.012	.192	.034
Relating & Struc.			.061		.137		.177		.082	.139	.154	.073
Concrete proc.			.156		.132		.069		.079	.078	.180	.058
Analysing			.067		.144		.239		.123	.214	.144	.109
Memorizing					.117		.232		.077	.181	.105	.070
Self‐Reg. Proc.	−.120		.165		.126		.189		.087	.140	.134	.064
Self‐Reg. Cont.	−.187		.185		.180		.153		.084	.059	.155	.040
Ext.‐Reg. Proc.		.116			.071		.232		.117	.213	.157	.117
Ext.‐Reg. Results	.183					.094	.211		.160	.234	.197	.158
Lack Regulation	−.178		.242		−.284	.219	−.111		.053	−.160	.081	.014

Note: *p* < .001 in left panel.

When restricting predictors to potential‐based goal pursuit, as shown in the right panel of Table 9, we observe that the best predicted learning patterns—External Regulation of both Process and Results—rely entirely on PAP as a predictor. Conversely, Critical Processing, Self‐Regulation of Content, and Lack of Regulation are weakly predicted by goal pursuit constructs, and this already modest predictive power is significantly reduced when predictors are limited to potential‐based goal facets.

### Achievement goals and module performance

The correlation matrix presented in Table 10 outlines the bivariate relationships between achievement goals and academic performance. An expanded version of Table 10 is included as Data S3, containing bootstrap 95% confidence intervals. Standard errors of all correlations fall in the [.010, .011] range, creating confidence intervals with a margin of error of about .021. An initial observation highlights the lack of substantial associations between performance and self‐based goals, whether approach or avoidance‐oriented. In contrast, task‐based, other‐based, and potential‐based goals, in both their forms, exhibit a positive correlation with performance. Despite earlier expectations from Elliot et al. (2015) suggesting that potential‐based goals might not predict performance effectively due to their abstract nature, this anticipation is not confirmed in our study's mastery‐based assessment context. Although the impact sizes are modest, it are the two facets of potential‐based goals, rather than those of self‐based goals, that represent the effect of intrapersonal goals on performance in the bivariate context. Interpersonal goals show a positive correlation with all performance categories but display a noticeable distinction between mathematics and statistics performance. Lastly, task‐based goals differentiate between the two types of performance: quizzes versus final exam.

**TABLE 10:** Correlations of achievement goal scale scores with module performance.

	TAP	TAV	SAP	SAV	OAP	OAV	PAP	PAV
Math exam	.137***	.091***	.015	.027**	.125***	.102***	.095***	.085***
Stats exam	.137***	.101***	−.004	.023*	.073***	.060***	.082***	.079***
Math quizzes	.093***	.072***	.042***	.052***	.122***	.109***	.100***	.071***
Stats quizzes	.100***	.078***	.041***	.058***	.090***	.085***	.105***	.078***

Note: *: *p* < .05; **: *p* < .01; ***: *p* < .001.

A structural equation model is utilized to explore multivariate relationships, employing cohort‐wise transformed performance scores. The left panel of Table 11 displays the standardized beta coefficients derived from this structural equation model. Particularly noteworthy is the absence of any avoidance‐oriented goal facets within the prediction model, and the reversal of roles for intrapersonal goal setting. In the multivariate analysis, self‐based goals, rather than potential‐based goals, show the largest beta coefficients. However, all beta coefficients of self‐based goals reverse in sign, compared to the bivariate case: in the multivariate context, when accounting for levels of task‐based and other‐based approach goals, self‐based approach goals negatively impact predicted performance, whereas potential‐based approach goals remain having a positive impact, but minor in size. Goals account for approximately 6% of the variation in the four performance categories, a small effect size.

**TABLE 11:** Standardized beta coefficients from the structural equation model predicting module performance from achievement goals; left panel applying all goal constructs, right panel potential‐based goals only.

	TAP	TAV	SAP	SAV	OAP	OAV	PAP	PAV	*R* ^2^	PAP	PAV	*R* ^2^
Math exam	.289		−.254		.139				.059	.072	.036	.010
Stats exam	.314		−.268		.092				.057	.067	.037	.009
Math quizzes	.254		−.248		.147		.079		.067	.127	.028	.022
Stats quizzes	.270		−.228		.084		.062		.055	.129	.019	.021

Note: *p* < .001 in left panel.

The limited contribution of potential‐based goal setting in predicting performance is also evident in the right panel of Table 11. Compared to the left panel, the explained variation drops significantly when task‐based and self‐based goal setting are excluded from the prediction equations.

## DISCUSSION

The descriptive analysis provides valuable insights into the internal and external validation of potential‐based goals. Correlations show that potential‐based goals are empirically distinct from other facets of goal‐setting, particularly from past‐based intrapersonal goals. Gender and prior education differences align with Martin's (2015) predictions, with higher levels of goal‐setting among female students and those with advanced prior education, with the most pronounced effects observed for gender differences.

Confirmatory factor models, both of first and second‐order type, affirm the distinctiveness of potential‐based goals as a latent factor separate from the other intrapersonal manifestations of achievement goals, the self‐based goals. However, potential‐based goals transcend mere categorization. Across various partial models focusing on the interplay between achievement goals and individual disposition instruments, potential‐based goals consistently emerge as a prominently articulated dimension, displaying noteworthy relationships with other learning dispositions. This prominence is evident in analyses examining the relationships between goal setting and cognitions and behaviours outlined in Martin's Motivation and Engagement Wheel. In all aspects of adaptive motivation and engagement, as well as maladaptive engagement, potential‐based goals exhibit a pronounced influence over task‐based goals, self‐based goals, and other‐based goals in their respective bivariate relationships. Furthermore, with the exception of Self‐belief, potential‐based approach goals take precedence in prediction equations across all facets of motivation and engagement in multivariate contexts. This consistent pattern extends to other dispositional variables such as implicit theories and associated effort beliefs.

Concerning learning mindsets and associated beliefs: our findings regarding multivariate prediction equations for incremental and entity theory, basically being mirror images, provide support to position taken in Boncquet et al. (2023), that the growth and fixed mindsets are entirely antithetical. In contrast, effort beliefs do not represent antithetical concepts, which aligns with the distinct structures of prediction equations for positive and negative effort beliefs.

A significant empirical observation is the limited explanatory power of achievement goals in understanding implicit theories. Structural equation models indicate that goal pursuit is only weakly associated with the incremental and entity views on learning, but has a much stronger connection with effort beliefs and learning motives, particularly the adaptive aspects: positive effort belief and autonomous motivation. Another notable finding is that approach dimensions tend to outweigh avoidance dimensions, with the exception being the two maladaptive concepts of Effort negative beliefs and Controlled motivation. These results underscore the pivotal role of effort beliefs in investigating the influence of mindsets on learning, consistent with previous studies by Lavrijsen et al. (2022), Boncquet et al. (2023), and Tempelaar, Rienties, Giesbers, and Gijselaers (2015).

An interesting aspect of this pattern relates to the placement of two intrapersonal goal dimensions: self‐based and potential‐based. Relationships involving potential‐based goals exhibit stronger associations than those of self‐based goals across various dimensions, including approach and avoidance tendencies, implicit theories, effort perspectives, and learning motives, both in bivariate and multivariate analyses. This aligns with hypotheses proposed by Martin (2015), suggesting that growth‐oriented goals are expected to have relatively robust associations with implicit theories. In our study, the effect size of the approach dimension of potential‐based goal setting in predicting positive effort beliefs is notably large, despite the overall effect size of the prediction equation remaining at a medium level.

The strong associations of potential‐based goal setting are most evident in its relationships with adaptive motivations and engagements, and the maladaptive engagements, applying the Motivation and Engagement Wheel framework. In all these relationships, prediction equations do not get beyond medium effect sizes, where the individual role of the approach dimension of potential‐based goal settings reaches large effect sizes (Self‐belief excerpted).

In exploring relationships with learning approaches, the recurring dominance of approach dimensions over avoidance dimensions is consistent, as observed in prior research (Vermunt & Donche, 2017). However, in contrast to previous studies, the significance of task‐based approach goals is overshadowed by the approach dimensions related to self, others, and potential‐based goal setting. This is to such an extent that, in the multivariate context, considering the effect of the other approach dimensions of goal setting, task‐based approach goals turn into negative predictors of self‐regulation and positive predictor of external regulation of learning.

In continental Europe, grading systems, criterion‐referenced and centred on conceptual mastery, contrast with the norm‐referenced grading systems common in Anglo‐Saxon classrooms. Within this context, approach goals emerge as more effective predictors of performance compared to avoidance goals, across all definitions of goals and in both univariate and multivariate relationships. Furthermore, among intrapersonal goals, potential‐based goals demonstrate stronger bivariate associations with performance than self‐based goals. In the multivariate context, the weak correlations of self‐based goal setting transform into negative beta coefficients, once the effects of task‐based and other‐based approach goals are accounted for. This is not a unique finding; Elliot et al. (2011) observed a similar phenomenon where self‐based goals, both approach and avoidance types, became negative predictors of performance when considering the effects of task‐based and other‐based goals. The positive bivariate relationships between self‐based goals and performance are likely due to collinearity among all goal types, as seen in Table 2. Collinearity, combined with positive correlations for task‐based and other‐based goals, results in positive correlations for self‐based goals. However, in the multivariate context, where the effect of self‐based goals is isolated from the indirect effects of other goal types, the remaining impact on performance is negative. This contradicts the expectation articulated in Elliot et al. (2015) that potential‐based goals would exhibit weaker correlations with performance due to their abstract nature. In our criterion‐referenced graded assessments, this hypothesis does not hold true in the bivariate context, whereas in the multivariate context, potential‐based goals are again preferred over self‐based goals as they retain the proper sign of the beta coefficients. Nevertheless, the predictive power remains limited, with task‐based approach goals accounting for most of this predictive capacity.

## LIMITATIONS

While the sample size is large, the dataset remains cross‐sectional. The inclusion of longitudinal data would have significantly enhanced the research process. Specifically, longitudinal data would have facilitated the differentiation between antecedence and consequence relationships, as demonstrated in Martin's study (2015). Additionally, the AGQ instrument uses examinations as a reference. In exploring student learning's growth orientation, a more expansive definition of achievement holds appeal. Personal‐best goals, as proposed by Martin (2011b), present an appealing alternative in this regard. Another limitation could be the composition of our sample. Due to the international nature of the university, our sample appears to include a more diverse range of nationalities compared to the US sample studied by Elliot et al. (2015) and the Australian sample studied by Martin (2015). However, European students are noticeably overrepresented. They function in a highly achievement‐oriented environment, though the European criterion‐referenced grading system tends to reduce competition among students compared to norm‐referenced grading systems.

## OVERALL CONCLUSIONS

In contrast to the concerns raised in Elliot et al. (2015), we did not detect significant collinearity in our goal questionnaire data nor any indication of response biases. The reason for this could be linked to both the prevailing grading system and the requirement for students to conduct a statistical analysis of their personal survey data, which likely encouraged them to offer truthful responses. Given this context, it becomes evident that employing a comprehensive 4X2 framework is entirely justified. However, if compelled to limit our framework to a 3X2 structure, the choice is unequivocally in favour of potential‐based goals. This decision is motivated not only by a desire to address an ‘overlooked area’ as noted by Elliot et al. back in 2015, and still remaining the case: in their review study of achievement motivation literature, Elliot and Sommet (2023) devote no more than a footnote to personal best goals and potential‐based goals. Our preference for including growth‐type goal setting behaviour in achievement motivation studies is primarily driven by their superior predictive capacity.

## AUTHOR CONTRIBUTIONS

**Dirk Tempelaar:** Conceptualization; investigation; writing – original draft; methodology; validation; visualization; writing – review and editing; formal analysis.

## CONFLICT OF INTEREST STATEMENT

The author declares no conflicts of interest.

## Supporting information


Data S1.

Data S2.

Data S3.

## Data Availability

The data that support the findings of this study are openly available in dataverse at https://dataverse.nl/.
