# Supplementary material for: The unrecognized potential of potential‐based achievement goals
Source: Br J Educ Psychol. 2024 Dec 14;95(2):421–45. doi: 10.1111/bjep.12728 (PMC12068019; doi:10.1111/bjep.12728)
Supplement: Supplementary file 1 — Data S1. [file BJEP-95-421-s002.docx]

AGQ extended instrument

Sources:

Elliot, A.J., Murayama, K., Kobeisy, A. & Lichtendfeld, S. (2015). Potential-based achievement goals, *British Journal of Educational Psychology*, *85*, 192-206. doi : 10.1111/bjep.12051

Elliot, A.J., Murayama, K., & Pekrun R. (2011). A 3 × 2 achievement goal model. *Journal of Educational Psychology*, *103*, 632-648, doi: 10.1037/a0023952

| Order | Name | Item |
| --- | --- | --- |
|  |  | Task-approach goal items |
| 1 | TAP1 | To get a lot of questions right on the exam and quizzes |
| 9 | TAP2 | To know the right answers to the questions on the exam and quizzes |
| 17 | TAP3 | To answer a lot of questions correctly on the exam and quizzes |
|  |  |  |
|  |  | Task-avoidance goal items |
| 2 | TAV1 | To avoid incorrect answers on the exam and quizzes |
| 10 | TAV2 | To avoid getting a lot of questions wrong on the exam and quizzes |
| 18 | TAV3 | To avoid missing a lot of questions on the exam and quizzes. |
|  |  |  |
|  |  | Self-approach goal items |
| 3 | SAP1 | To perform better on the exam and quizzes than I have done in the past on these types of test |
| 11 | SAP2 | To do well on the exam and quizzes relative to how well I have done in the past on such tests. |
| 19 | SAP3 | To do better on the exam and quizzes than I typically do in this type of situation. |
|  |  |  |
|  |  | Self-avoidance goal items |
| 4 | SAV1 | To avoid doing worse on the exam and quizzes than I normally do on these types of tests. |
| 12 | SAV2 | To avoid performing poorly on the exam and quizzes compared to my typical level of performance. |
| 20 | SAV3 | To avoid doing worse on the exam and quizzes than I have done on prior tests of this type. |
|  |  |  |
|  |  | Other-approach goal items |
| 5 | OAP1 | To outperform other students on the exam and quizzes. |
| 13 | OAP2 | To do well compared to others in the group on the exam and quizzes. |
| 21 | OAP3 | To do better than my peers on the exam and quizzes. |
|  |  |  |
|  |  | Other-avoidance goal items |
| 6 | OAV1 | To avoid doing worse than other students on the exam and quizzes |
| 14 | OAV2 | To avoid doing poorly in comparison to others on the exam and quizzes. |
| 22 | OAV3 | To avoid performing poorly relative to my peers on the exam and quizzes |
|  |  |  |
|  |  | Potential-approach goal items |
| 7 | PAP1 | To do as well as I can possibly do on the exam and quizzes. |
| 15 | PAP2 | To do the best that I can do on the exam and quizzes. |
| 23 | PAP3 | To do my own personal best on the exam and quizzes. |
|  |  |  |
|  |  | Potential- avoidance goal items |
| 8 | PAV1 | To avoid doing poorly in comparison to my absolute best on the exam and quizzes. |
| 16 | PAV2 | To avoid doing worse than I know I can do on the exam and quizzes. |
| 24 | PAV3 | To avoid doing worse than my very best on the exam and quizzes. |
