# Supplementary material for: The unrecognized potential of potential‐based achievement goals
Source: Br J Educ Psychol. 2024 Dec 14;95(2):421–45. doi: 10.1111/bjep.12728 (PMC12068019; doi:10.1111/bjep.12728)
Supplement: Supplementary file 2 — Data S2. [file BJEP-95-421-s003.docx]

MES instrument

Source:

Martin, A. J. (2011). The Motivation and Engagement Scale (5th ed.). Sydney, NSW: Lifelong Achievement Group.

| Scale | Sample-item |
| --- | --- |
| Self-belief | If I try hard, I believe I can do my university work well |
| Valuing school | Learning at university is important |
| Learning focus | I feel very pleased with myself when I really understand what I’m taught at university |
| Planning | Before I start an assignment, I plan out how I am going to do it |
| Study management | When I study, I usually study in places where I can concentrate |
| Persistence | If an assignment is difficult, I keep working at it trying to figure it out |
| Learning anxiety | When exams and assignments are coming up, I worry a lot |
| Failure avoidance | Often the main reason I work at university is because I don’t want people to think bad things about me |
| Uncertain control | When I get a good mark I’m often not sure how I’m going to get that mark again |
| Self-sabotage | Sometimes I don’t try hard at assignments so I have an excuse if I don’t do so well. |
| Disengagement | I’ve pretty much given up being involved in things at university |

Mindset instrument

Sources:

Dweck, C. S. (1999). Self-theories: Their role in motivation, personality, and development. Philadelphia: Psychology Press.

Blackwell, L. S. (2002). Psychological mediators of student achievement during the transition to junior high school: The role of implicit theories. Unpublished doctoral dissertation, Columbia University, New York.

| Scale | Sample-item |
| --- | --- |
| Entity theory | You have a certain amount of intelligence, and you can’t really do much to change it |
| Incremental theory | No matter who you are, you can significantly change your intelligence level |
| Effort negative | If you have to work hard on some problems, you’re probably not very good at them |
| Effort positive | When you’re good at something, working hard allows you to really understand it |

Academic Motivation instrument

Source:

Vallerand, R. J., Pelletier, L. G., Blais, M. R., Brière, N. M., Senécal, C., and Vallières, E. F. (1992). The Academic Motivation Scale: A Measure of Intrinsic, Extrinsic, and Amotivation in Education. Educational and Psychological Measurement, 52, 1003–1017. doi: 10.1177/0013164492052004025

| Scale | Sample-item, all with stem: Why do you go to college? |
| --- | --- |
| Intrinsic to Know | Because I experience pleasure and satisfaction while learning new things. |
| Intrinsic Accomplishm | For the pleasure I experience while surpassing myself in my studies |
| Intrinsic Stimulation | For the intense feelings I experience when I am communicating my own ideas to others |
| Extrinsic Identified | Because I think that a college education will help me better prepare for the career I have chosen. |
| Extrinsic Introjected | To prove to myself that I am capable of completing my college degree |
| Extrinsic External | Because with only a high-school degree I would not find a high-paying job later on |
| A-motivation | Honestly, I don't know; I really feel that I am wasting my time in school |

Learning patterns instrument

Source:

Vermunt, J. D. (1998). The regulation of constructive learning processes. British Journal of Educational Psychology, 68, 149–171. doi: 10.1111/j.2044-8279.1998.tb01281.x

| Scale | Sample-item |
| --- | --- |
| Critical Proc. | I compare my view of a topic in the course with the view of the authors of the book being dealt with |
| Relating & Struc. | I find out what the similarities and differences are between the theories which are dealt with in a course |
| Concrete proc. | I try to interpret events in everyday reality using the knowledge which I have gained in a course |
| Analysing | I work through a chapter in a text book point by point and study each finished section separately |
| Memorising | I repeat the most important parts of the material until I know them by heart |
| Self-Reg. Proc. | In order to test my progress in learning, I try, after studying the textbook, to formulate the main points in my own words |
| Self-Reg. Cont. | In addition to the textbook I also study other books/articles which relate to the content of the course |
| Ext.-Reg. Proc. | If a textbook contains questions or assignments, I work them out completely as soon as I come across them while studying |
| Ext.-Reg. Results | I test my progress purely by answering the questions and doing the assignments and exercises in the textbook that the teacher tells us to do |
| Lack Regulation | I realise that it is not clear to me what I need to remember and what I do not need to remember |
